# Comparison of the Biological and Chemical Synthesis of Schwertmannite at a Consistent Fe^2+^ Oxidation Efficiency and the Effect of Extracellular Polymeric Substances of *Acidithiobacillus ferrooxidans* on Biomineralization

**DOI:** 10.3390/ma11091739

**Published:** 2018-09-15

**Authors:** Yongwei Song, Yelin Liu, Heru Wang

**Affiliations:** School of Information and Safety Engineering, Zhongnan University of Economics and Law, Wuhan 430073, China; liuyeline@163.com (Y.L.); Z0004382@zuel.edu.cn (H.W.)

**Keywords:** *Acidithiobacillus ferrooxidans*, H_2_O_2_, schwertmannite, extracellular polymeric substances, biomineralization

## Abstract

Schwertmannite is an environmental mineral material that can promote the natural passivation of heavy metal elements, thereby reducing environmental pollution from toxic elements. However, the fundamental reason for the difference between the chemically (H_2_O_2_-FeSO_4_) and biologically (*Acidithiobacillus ferrooxidans*-FeSO_4_) synthesized schwertmannite is still unclear. In this study, X-ray diffraction, scanning electron microscopy, the Brunauer–Emmett–Teller method, and X-ray fluorescence spectrometry were used to compare the structure, specific surface area, and elemental composition of schwertmannite synthesized by biological and chemical methods. The removal capacity of As(III) by the two kinds of schwertmannite and the effects of extracellular polymeric substances (EPS) on biogenetic schwertmannite were also investigated. At a consistent Fe^2+^ oxidation efficiency, the chemical method synthesized more schwertmannite than the biological method over a 60-h period. The biosynthesized schwertmannite had a “chestnut shell” shape, with a larger particle size and specific surface than the chemically synthesized schwertmannite, which was relatively smooth. The saturated adsorption capacities of the biologically and chemically synthesized schwertmannite were 117.0 and 87.0 mg·g^−1^, respectively. After exfoliation of the EPS from *A. ferrooxidans*, the biosynthesized schwertmannite displayed a “wool ball” shape, with rough particle surfaces, many microporous structures, and a larger specific surface area. The schwertmannite yield also increased by about 45% compared with that before exfoliation, suggesting that the secretion of EPS by *A. ferrooxidans* can inhibit the formation of schwertmannite.

## 1. Introduction

Schwertmannite is a widely used adsorbent material with a chemical composition of Fe_8_O_8_(OH)_8−2x_(SO_4_)_x_ (1≤ x ≤1.75) [1]. Schwertmannite particles are nanometer-sized and have an irregular pore structure that contains large numbers of groups such as –OH and SO_4_^2−^ [2,3]. Its specific surface area is generally from 100 to 200 m^2^g^−1^. The SO_4_^2−^ groups in the mineral’s pores can be replaced by anions with similar ionic radii (PO_4_^3−^, CrO_4_^2−^, etc.), which allows schwertmannite to adsorb or coprecipitate toxic and harmful elements in water. This process can be used to remove heavy metal (metalloid) ions from wastewater [4,5]. Many studies have also shown that schwertmannite can promote the natural passivation of heavy metal (metalloid) elements, thereby reducing or eliminating the environmental pollution from toxic elements, which is of great significance to environmental regulations [3,6,7,8,9,10].

Schwertmannite is ubiquitous in acidic sulfate environments, but naturally formed schwertmannite tends to have a low purity and is usually doped with other mineral phases (such as jarosite, ferrihydrite, goethite, etc.), meaning that it is difficult to purify and use for environmental applications. Significant research has, therefore, been conducted into the efficient and rapid synthesis of schwertmannite. The synthetic pathways for schwertmannite mainly include biological and chemical methods. The biological methods generally involve using *Acidithiobacillus ferrooxidans* (*A. ferrooxidans*) to perform the catalytic oxidation of FeSO_4_ [2,6,11], whereas the chemical methods include either Fe^3+^ hydrolysis or Fe^2+^ oxidation [4,11]. Reactions (1)–(2) present the schwertmannite formation processes [6]:

4Fe^2+^ + O_2_ + 4H^+^ → 4Fe^3+^ + 2H_2_O (1)

8Fe^3+^ + 14H_2_O + SO_4_^2−^ → Fe_8_O_8_(OH)_6_SO_4_ (ideal schwertmannite) + 22H^+^ (2)

Studies have shown that slowly adding H_2_O_2_ dropwise to the FeSO_4_ solution, stirring for 24 h, centrifuging, and freeze-drying can increase the purity of the schwertmannite [4,12]. Li et al. [13] compared the appearance features of schwertmannite synthesized by both chemical (H_2_O_2_-FeSO_4_) and biological methods (*A. ferrooxidans*-FeSO_4_), finding that the total Fe precipitation efficiencies of the chemical and biological systems were 43.1% and 36.7%, respectively. They also found that the biosynthesized schwertmannite was relatively more uniform and dispersed, and the specific surface area of the biosynthesized schwertmannite was much higher (45.63 m^2^·g^−1^) than that of the chemically synthesized schwertmannite (3.17 m^2^·g^−1^). However, their chemical method involved adding a sufficient amount of H_2_O_2_ to rapidly oxidize FeSO_4_ (oxidation time <1 h), before transferring it into a shaker to continue the mineralization reaction. In contrast, their biological catalytic oxidation process was mild and long-lasting (oxidation time 12 h). So, the reason for the differences in the characteristics of the minerals is not clear. In order to investigate the effect of FeSO_4_ oxidation efficiency on the synthesis of schwertmannite, Liu et al. [14] conducted slow, medium, and rapid oxidation processes of FeSO_4_ by controlling the frequency of equal addition of H_2_O_2_ (oxidation time 2–50 h). Their results showed that while a low H_2_O_2_ supply rate obviously inhibited the synthesis of schwertmannite, it also significantly increased its specific surface area. Yet, in previous comparisons of chemical and biological methods for synthesizing schwertmannite, the important control factor—a consistent Fe^2+^ oxidation efficiency—was neglected, which is crucial for the comparison and analysis of the mineralization characteristics of the two methods.

Li et al. [13] found that the chemically synthesized schwertmannite particle was finely spherical in shape, with a smooth surface and a particle size of 400–600 nm. In comparison, the biosynthesized schwertmannite that they produced had a particle size of 2~3 μm and exhibited a typical “sea urchin” needle burr. The reason for the differences between the two was not immediately apparent, however. It has been reported that biomineralization includes two pathways. The first, biologically induced mineralization, involves the microbial secretion of metabolites (extracellular polymeric substances, referred to as EPS) including polysaccharides, organic acids, and peptide compounds. This approach may lead to the aggregation of mineral particles. The second pathway is biologically controlled mineralization, in which microbes play a significant role in controlling mineral nucleation and growth, and the synthesized minerals are often nanometer- or micron-level-sized [15]. Banfield et al. [16] posited that the EPS secreted by microorganisms may have a certain regulatory effect on the surface structure of minerals, which could be used as a template for the concentration, aggregation, and mineralization of ions to form crystal cores. Other studies have highlighted that EPS can promote the adhesion of microorganisms onto the surface of mineral particles, which could hinder the further polymerization of minerals [17]. In addition, EPS easily form a complex of EPS–Fe^3+^ with Fe^3+^, meaning that the partial oxidation product (Fe^3+^) cannot participate in the reaction, which in turn affects the synthesis of minerals [18]. In summary, though there have been many studies into the effects of *A. ferrooxidans* on the secretion of EPS, their conclusions are inconsistent.

As highlighted above, the reasons for the differences between the biological and chemical synthesis of schwertmannite are still unclear, and it remains to be verified whether different oxidation rates of FeSO_4_ and/or the secretion of EPS by *A. ferrooxidans* play decisive roles in the mineralization process (promotion or inhibition). Therefore, the objectives of the present work were to (1) evaluate whether there are differences in the characteristics of schwertmannite synthesized by biological and chemical methods under the same Fe^2+^ oxidation efficiency; (2) compare the removal capacity of As(III) by the two kinds of schwertmannite as adsorbents; and (3) investigate the influence mechanism of extracellular polymeric substances on the biosynthesis of schwertmannite.

## 2. Materials and Methods

### 2.1. Materials

The modified 9 K medium used in this study contained (NH_4_)_2_SO_4_ (3.5 g), KCl (0.119 g), K_2_HPO_4_ (0.058 g), Ca(NO_3_)_2_·4H_2_O (0.0168 g), and MgSO_4_·7H_2_O (0.583 g), dissolved into 100 mL of deionized water. This medium, which had a pH of 2.5, was then autoclaved at 121 °C for 30 min.

*A. ferrooxidans* resting cells: *A. ferrooxidans* LX5 (CGMCC No. 0727), obtained from the China General Microbiological Culture Collection Center (CGMCC, Beijing, China). *A. ferrooxidans* were inoculated in the modified 9K medium (containing 44.48 g·L^−1^ FeSO_4_·7H_2_O), and were shaken in a 180-rpm shaker at 28 °C. The culture was stopped at the end of the exponential growth phase. Subsequently, each culture was initially filtered (using qualitative filter paper) to remove the precipitate, and the filtrates were centrifuged at 3000 g for 10 min at 4 °C to precipitate the bacterial cells. After being washed and centrifuged in a H_2_SO_4_ solution (pH 1.5) three times to remove various adsorbed or doped ions, the cells were resuspended in a further H_2_SO_4_ solution (pH 2.5), referred to herein as the *A. ferrooxidans* resting cells [19].

### 2.2. Experimental Setting

#### 2.2.1. Comparison of the Biological and Chemical Synthesis of Schwertmannite at a Consistent Fe^2+^ Oxidation Efficiency

Biological synthesis of schwertmannite: In a 500 mL Erlenmeyer flask, the *A. ferrooxidans* resting cells were added to 225 mL of a solution containing 11.12 g of FeSO_4_·7H_2_O (the additive content of FeSO_4_·7H_2_O was referenced to the literature [14]). The *A. ferrooxidans* resting cells inoculum was 10% (V/V), and the density was 6.0 × 10^6^ cell·mL^−1^. The system was buffered to a pH of 3.0 using 1:1 H_2_SO_4_, before being placed in a shaker at 28 °C, 180 rpm, to achieve biomineralization [13]. All experiments were performed in triplicate. During the mineralization process, approximately 1 mL of the incubated mixture was periodically sampled and filtered through membranes (0.22 μm in pore size) to monitor and calculate the concentration of Fe^2+^, the oxidation efficiency of Fe^2+^, and the precipitation efficiency of the total Fe. The fit of the oxidation efficiency of Fe^2+^ and the culture time were then used to calculate the average bio-oxidation efficiency of Fe^2+^ throughout the whole reaction process. After the complete oxidation of Fe^2+^, shaking was stopped, and the synthesized schwertmannite was collected on medium-speed qualitative filter paper.

Chemical synthesis of schwertmannite: Preliminary tests showed that a volume of ~6 mL of 30% H_2_O_2_ is required to fully oxidize 11.12 g of FeSO_4_·7H_2_O. Therefore, 11.12 g of FeSO_4_·7H_2_O was dissolved in an Erlenmeyer flask with an effective volume of 250 mL, and the pH of the system was buffered to 3.0 using 1:1 H_2_SO_4_. The input flow rate of H_2_O_2_ was set according to the average Fe^2+^ oxidation efficiency of the schwertmannite, as determined during the biological method. The Erlenmeyer flask was continuously shaken in a shaker at 28 °C, 180 rpm [14]. Each treatment process was conducted in triplicate. At the start of the reaction, H_2_O_2_ was pumped into the above system through a peristaltic pump (HL–2B), according to the H_2_O_2_ input flow rate, to simulate a continuous bio-oxidation mineralization process. To test the degree of synchronization between the chemical and biological oxidation processes, approximately 1 mL of the incubated mixture was also periodically sampled, in the same way as described above for the biological method, and the synthesized schwertmannite was collected at the end of the culture, again as described above.

#### 2.2.2. Comparison of the Biological and Chemical Synthesis of Schwertmannite on As(III) Adsorption

To compare the As(III) adsorption removal efficiency of the schwertmannite obtained under different conditions, experiments were conducted by adding 25 mg of synthesized schwertmannite to 250-mL Erlenmeyer flasks each containing 100 mL of solution, initially containing 0–30 mg·L^−1^ As(III) (prepared from As_2_O_3_). The suspension pH was adjusted to 7.5 by adding 1 mol·L^−1^ HCl or NaOH. All Erlenmeyer flasks were shaken in a shaker at 28 °C and 180 rpm [2]. Each treatment process was conducted in triplicate. After 4 h, the sampled suspension was filtered through 0.22 μm membranes and the As(III) concentration in the liquid phases were examined. The As(III) adsorption capacity was subsequently calculated according to the following formula:

As(III) adsorption capacity (mg·g^−1^) = (C_0_ − C_t_) × 0.10 L/(25 mg × 10^−3^), where C_0_ is the initial As(III) concentration (mg·L^−1^) and C_t_ is the As(III) concentration (mg·L^−1^) in the filtrate at different times. 0.10 L is the effective volume of the solution and 25 mg is the weight of schwertmannite.

#### 2.2.3. Comparison of Schwertmannite before and After Stripping of the EPS 

Three Erlenmeyer flasks were prepared, each with an effective volume of 225 mL and each containing 11.12 g of FeSO_4_·7H_2_O. 25 mL of the *A. ferrooxidans* resting cells were added to these flasks, treated in one of three ways. Treatment one contained conventional *A. ferrooxidans* resting cells (herein referred to as *A. f*); for treatment two, the *A. ferrooxidans* resting cells were centrifuged at 20,000 g for 20 min at 4 °C to exfoliate the EPS [20], and were then shaken again to keep the *A. ferrooxidans* and EPS in suspension, thereby obtaining *A. ferrooxidans* resting cells with the EPS exfoliated but not removed (herein referred to as *A. f*+EPS); and for treatment three, the *A. ferrooxidans* resting cells were centrifuged at 20,000 g for 20 min at 4 °C, then the supernatant (containing EPS) was removed. For this final treatment, the same volume of deionized water was used to resuspend *A. ferrooxidans*, obtaining *A. ferrooxidans* resting cells where the EPS had been both exfoliated and removed (herein referred to as *A. f*−EPS). The cell densities of the different types of *A. ferrooxidans* in the above system were detected to be about 6.0 × 10^6^ cell·mL^−1^. The pH of the system was buffered to 3.0 using 1:1 H_2_SO_4_, then the EPS content was determined in supernatant (filtered through 0.22 membranes). All Erlenmeyer flasks were shaken in a shaker at 28 °C and 180 rpm. The Fe^2+^ concentration and Fe^2+^ oxidation efficiency were sampled and calculated in solution according to 2.2.1. After culture for 72 h, the synthesized schwertmannites were harvested by filtering through a Whatman No. 4 filter paper and were then washed once with distilled water. Subsequently, the minerals were dried at 60 °C to constant weight.

### 2.3. Analytical Procedures

The solution pH was measured using a pHS-3C digital pH meter with a resolution of 0.01 pH unit. The Fe^2+^ and total Fe concentrations were determined using the 1,10-phenanthroline method according to the standard method [21], then the Fe^2+^ oxidation efficiency and total Fe precipitation efficiency were calculated according to the following formulas:

Fe^2+^ oxidation efficiency (%) = [(C_Fe(II)_ − C’_Fe(II)_)/C_Fe(II)_] × 100%, where C_Fe(II)_ is the initial Fe^2+^ concentration (mg·L^−1^), and C’_Fe(II)_ is the Fe^2+^ concentration (mg·L^−1^) at different times.

Total Fe precipitation efficiency (%) = [(C_total Fe_ − C’_total Fe_)/C_total Fe_] × 100%, where C_total Fe_ is the initial total Fe concentration (mg·L^−1^), and C’_total Fe_ is the total Fe concentration (mg·L^−1^) at different times. The weight of the precipitate was measured using an electronic balance (EL204); The specimen of the mineral sample was produced by the tabletting method, then the mineral phase of the sample was determined via X-ray diffraction (XRD, Bruker D8A25, Bruker Corporation, Karlsruhe, Germany) using CuKα radiation (40 kV, 40 mA); the samples were scanned from 10 to 80° 2*θ* with a step increment of 0.01° 2*θ* and 6°·min^−1^ scanning speed. Adhesive double-sided glue was applied to the tray, then the sample was fixed onto the double-sided glue and blown gently with a rubber balloon to make it evenly distributed, scanning electron microscopy (SEM) was performed using a Hitachi SU8010 (Hitachi Limited, Tokyo, Japan). The specific surface areas of the secondary iron minerals were determined using the Brunauer-Emmett-Teller (BET) adsorption method (TriStar II 3020, Micromeritics Instrument Corp, Norcross, GA, USA) and N_2_ as the adsorbate [19]. The total As concentrations in the solution were analyzed through atomic fluorescence spectroscopy (AFS-9730, Beijing Haiguang Instrument Co., Ltd, Beijing, China) with a detection limit of 0.01 μg·L^−1^ [22]. The exfoliated EPS content in the supernatant was analyzed for total organic carbon (TOC) by using a TOC analyzer (multi N/C 3100, Analytik Jena AG, Jena, Germany). The particle sizes of the samples were obtained by a laser particle size analyzer (Microtrac S3500, Microtrac Inc., Montgomeryville, PA, USA), the samples were ultrasound-assisted dispersed in water and then transported towards the measuring cell. The dried sample was placed in a measuring cup to measure the mineral elemental composition using an X-ray fluorescence spectrometer (XRF-1800, Shimadzu, Tokyo, Japan), and the molar ratio of Fe to S in the synthesized minerals was calculated using this XRF data.

## 3. Results and Discussion

### 3.1. Comparison of Fe^2+^ Oxidation Efficiency and the Total Fe Precipitation Efficiency in Biological Versus Chemical Mineralization Systems

**Figure 1:**
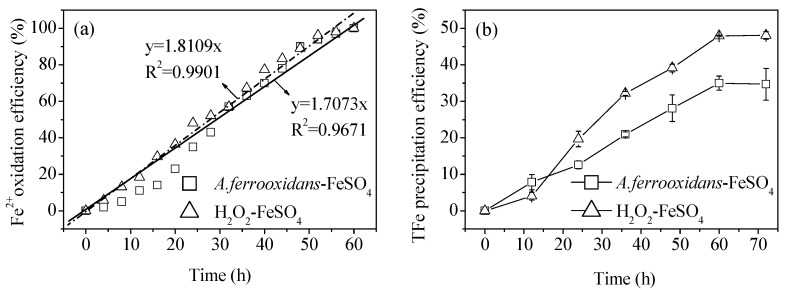
Comparison of (**a**) Fe^2+^ oxidation efficiency and (**b**) total Fe precipitation efficiency in the biological and chemical mineralization systems at 28 °C and 180 rpm for 72 h. Experimental conditions: initial pH = 3.0; FeSO_4_·7H_2_O dosage 11.12 g; *A. ferrooxidans* density 6.0 × 10^6^ cell·mL^−1^; H_2_O_2_ volume 6 mL.

Figure 1a shows the bio-oxidation of an 8.96 g·L^−1^ Fe^2+^ solution and simulates the actual oxidation efficiency of Fe^2+^ measured at the equivalent level of chemical oxidation (H_2_O_2_), according to the average Fe^2+^ bio-oxidation efficiency. *A. ferrooxidans* showed an adaptation period at the initial stage of inoculation, suggesting that it was affected by the environment. The bio-oxidation efficiency of Fe^2+^ was low. After 24 h, it entered a linear oxidation trend, and all Fe^2+^ had been oxidized after 60 h. The bio-oxidation efficiency of Fe^2+^ was significantly positively correlated with the reaction time, and the average oxidation rate of Fe^2+^ per unit time was 0.15 g·(L·h)^−1^. Compared with the research results of Li et al. [13], the Fe^2+^ bio-oxidation process was relatively slow (the complete oxidation time node was delayed 48 h). The reason for this is likely because the Fe^2+^ concentration in this study increased from 4.48 g·L^−1^ to 8.96 g·L^−1^, and the *A. ferrooxidans* inoculation density decreased from 2.0 × 10^7^ cell·mL^−1^ to 6.0 × 10^6^ cell·mL^−1^. In the H_2_O_2_-FeSO_4_ chemical oxidation system, the actual value of the Fe^2+^ chemical oxidation efficiency (measured by uniformly adding H_2_O_2_ according to the biological method Fe^2+^ oxidation efficiency index) was basically consistent with the theoretical calculation value, which also indirectly confirmed that the control of the equivalent oxidation efficiency of Fe^2+^ in this study was effective.

Figure 1b shows the dynamic change of the total Fe precipitation efficiency during both the biological and chemical mineralization processes. During these processes, alongside the continuous oxidation of Fe^2+^, the oxidation product, Fe^3+^, was continuously being hydrolyzed to synthesize schwertmannite. The experiment highlighted an obvious difference in the changing trends of the total Fe precipitation efficiency between the biological and chemical mineralization systems, at almost identical Fe^2+^ oxidation efficiency values. In agreement with the results of previous studies [13,14], the mineral yield of the chemical method was higher than that of the biological method, as illustrated by the fact that the total Fe precipitation efficiencies in the solutions after 60 h of reaction were 47.9% and 35.0%, respectively. Reactions (1)–(2) clearly show that the oxidation of Fe^2+^ involves a two-step acid effect process. The first step is the acid-depleting oxidation of Fe^2+^, while the second step is the acid-producing hydrolysis of Fe^3+^ to form schwertmannite. Therefore, after the Fe^2+^ oxidation (consistent efficiency) and the Fe^3+^ hydrolysis, the final pH of the reaction system should be controlled by the reaction (2). In this case, the higher efficiency of Fe^3+^ hydrolysis, the more H^+^ can be released to lower the pH of the solution; the pH was determined by the total Fe precipitation efficiency. So, the medium pH is not a significant factor for lowering of total Fe precipitation efficiency in the two reaction systems. This also indirectly indicates that the viewpoint adopted in previous studies, in which the difference between the chemical and biological synthesis of schwertmannite was attributed to differences in the Fe^2+^ oxidation efficiency (i.e., Fe^3+^ supply efficiency), has a certain one-sidedness. It is worth noting that in the initial stage of the reaction (12 h), the bio-oxidation efficiency of Fe^2+^ was much lower (11.0%) than that of the chemical oxidation (18.4%; Figure 1a), but the biological method was more conducive to the synthesis of schwertmannite in this period. For instance, the total Fe precipitation efficiencies of the biological and chemical methods were 7.8% and 4.0%, respectively. Their results showed that *A. ferrooxidans* played an inductive role in controlling mineral nucleation and growth. Under the unfavorable conditions of a low initial supply efficiency of Fe^3+^, the bacteria itself could have been used as seed crystal to shorten the induction period of mineral synthesis and stimulate Fe^3+^ (which was at low concentrations) to rapidly synthesize and precipitate [15,23,24]. Furthermore, the EPS secreted by the surface of *A. ferrooxidans* are also known to provide templates for the polymerization and mineralization of ions, promoting the efficient synthesis of minerals [25,26]. A study by Chan et al. [27] showed that the glial acidic polysaccharide component that secretes EPS on the cell surface can induce and regulate akageneite crystals to form fibrous filamentous morphological structures.

In general, the more seeds, the more favorable the environment was for the induction of mineral synthesis [23]. However, when the mineralization time exceeded 12 h, the biosynthesis of the schwertmannite appears to have been inhibited. There may be two reasons for this inhibition. Firstly, when large volumes of the mineral are synthesized, the EPS secreted by *A. ferrooxidans* adhere easily to the surface of the new minerals. This could have caused agglomeration, weakening the stimulation induction ability of the minerals, and thereby reducing the total Fe precipitation efficiency of the solution [23]. The chemical method differed from the biological method at this time because during the initial reaction stage of the H_2_O_2_-FeSO_4_ system, the main reaction was Fe^2+^ oxidation. Once Fe^3+^ was generated (the Fe^2+^ oxidation product), it began to hydrolyze into minerals, and later synthesis then accelerated due to the stimulation and induction of the seed crystals of the new minerals. Secondly, the mineralization process can be summarized as Fe^2+^→Fe^3+^→minerals, and the intermediate Fe^3+^ step plays a decisive role in the subsequent mineralization. The EPS secreted by *A. ferrooxidans* may form a complex with Fe^3+^ (EPS–Fe^3+^) which in turn may prevent Fe^3+^ from participating in the mineralization process, thus inhibiting the synthesis of minerals [18].

### 3.2. The XRD Patterns and SEM Images of the Biologically and Chemically Synthesized Schwertmannite

**Figure 2:**
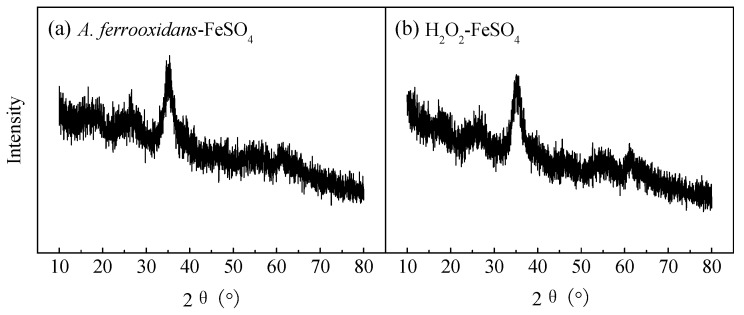
XRD patterns of (**a**) biologically and (**b**) chemically synthesized schwertmannite at 28 °C and 180 rpm for 72 h. Experimental conditions: initial pH = 3.0; FeSO_4_·7H_2_O dosage 11.12 g; *A. ferrooxidans* density 6.0 × 10^6^ cell·mL^−1^; H_2_O_2_ volume 6 mL.

XRD is the most effective method to distinguish crystalline and amorphous minerals and identify mineral types. Figure 2 shows two XRD patterns of the schwertmannite collected from the end cultures of the biological (Figure 2a) and chemical (Figure 2b) culture. Referring to the Joint Committee on Powder Diffraction Standards (JCPDS) XRD pattern of amorphous schwertmannite (No: 47-1775) [28], the diffraction peak positions and relative intensities of the two minerals (2*θ* = 18.24, 26.27, 35.16, 39.49, 46.53, 55.29, 61.34, 63.69°) were consistent with the standard schwertmannite; no other characteristic diffraction peaks were found. These two minerals were identified as a single mineral phase.

**Figure 3:**
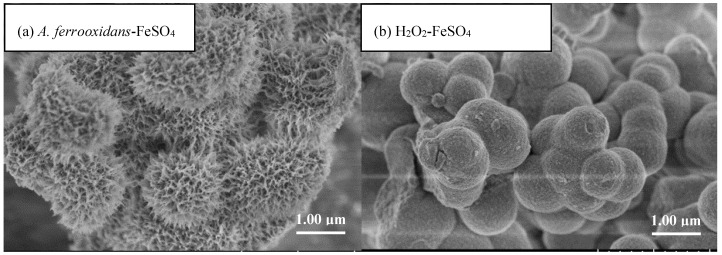
SEM images of (**a**) biologically and (**b**) chemically synthesized schwertmannite at 28 °C and 180 rpm for 72 h. Experimental conditions: initial pH = 3.0; FeSO_4_·7H_2_O dosage 11.12 g; *A. ferrooxidans* density 6.0 × 10^6^ cell·mL^−1^; H_2_O_2_ volume 6 mL.

The appearance features, such as mineral crystallinity, particle size, and agglomeration of the minerals were visually inspected by SEM [29]. Figure 3 shows the SEM images of schwertmannite collected from the two methods. The minerals from both methods were spherical-to-ellipsoidal in shape but had significantly different structural appearances. The surface of the chemically synthesized schwertmannite was relatively smooth; the particles were bonded and agglomerated with each other, and new spherical particles covered the surface of the schwertmannite particle agglomerate, which was consistent with the apparent characteristics of the chemically synthesized schwertmannite reported by Ran and Yu [30]. The particle median diameter was ~1.34 μm, and the specific surface area was 6.31 m^2^·g^−1^, which was within the range of 4–14 m^2^·g^−1^ reported by Regenspurg et al. [4]. The appearance structure of the biosynthesized schwertmannite was significantly different. Agglomeration was obvious, the particles exhibited “chestnut shell” and “sea urchin” shapes, and the mineral surfaces were covered with needle-like burrs. The pores were large, and the particle median diameter was ~1.52 μm, with a specific surface area of 58.79 m^2^·g^−1^. Previous studies have suggested that agglomeration may occur due to the secretion of the polysaccharides, organic acids, and peptide compounds produced by *A. ferrooxidans*; these compounds have a strong modification effect on mineral surfaces [16]. In addition, Loan et al. [11,31,32,33] found that schwertmannite formed in an acidic mine environment dominantly displayed a spherical “sea urchin”-like structure, which had a diameter of only 300–500 nm, however, and its surface was covered with 60–90 nm-sized needle-like burrs.

### 3.3. Comparison of the Biological and Chemical Synthesis of Schwertmannite on As(III) Adsorption

**Figure 4:**
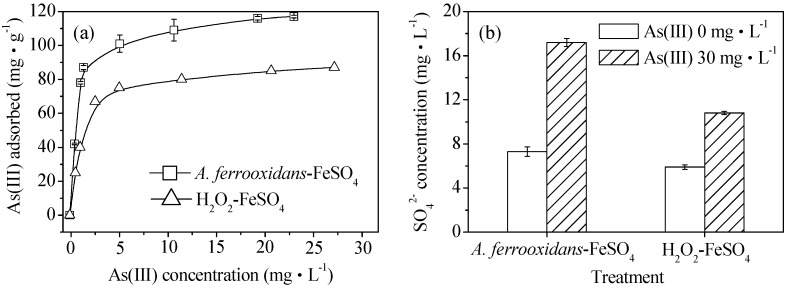
(**a**) As(III) adsorption capacity and (**b**) SO_4_^2−^ variation during As(III) adsorption on biologically and chemically synthesized schwertmannite at 28 °C and 180 rpm for 4 h. Experimental conditions: initial pH = 7.5; As(III) concentration 0–40 mg·L^−1^; schwertmannite 0.25 g·L^−1^.

The isothermal adsorption curves of As(III) obtained by the biological and chemical synthesis of schwertmannite at 28 °C are shown in Figure 4a. The saturated adsorption capacities of the biologically and chemically synthesized schwertmannite were 117.0 and 87.0 mg·g^−1^, respectively. The maximum adsorption capacity of the biogenic schwertmannite in this experiment is greater than that of activated alumina, at 3.48 mg·g^−1^ [34] and iron-based adsorbents with a maximum adsorption capacity of 113.9 mg·g^−1^ have also been reported recently [2]. As shown in Figure 4b, the SO_4_^2−^ concentration was 5.9–7.3 mg·L^−1^ in the As(III)-free schwertmannite suspension throughout the experiment. However, the SO_4_^2−^ concentration in the biologically or chemically synthesized schwertmannite suspension of 0.25 g·L^−1^ increased from the initial 7.3 or 5.9 mg·L^−1^ to 17.2 or 10.8 mg·L^−1^ after As(III) adsorption for 4 h. It is interesting to note that the release kinetic of SO_4_^2−^ is found to be well in accordance with that of the As(III) adsorption on schwertmannite, suggesting that exchange reactions between the SO_4_^2−^ and As(III) species occur during As(III) adsorption onto schwertmannite. These results are in good agreement with those of Burton et al. [35] who found that As(III) could be incorporated into the schwertmannite structure by exchanging for the tunnel SO_4_^2−^.

### 3.4. Effects of A. ferrooxidans on Schwertmannite Synthesis before and after Stripping of the EPS

**Figure 5:**
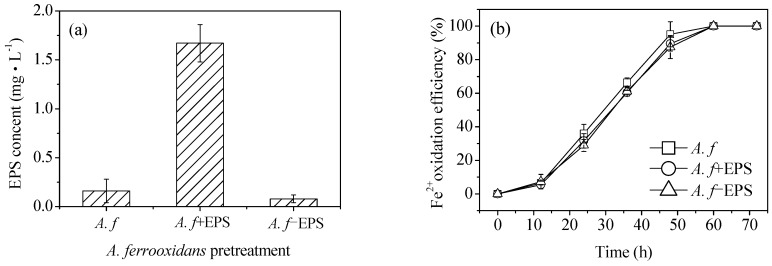
Comparison of (**a**) EPS content in supernatant and (**b**) Fe^2+^ oxidation efficiency under different treatment at 28 °C and 180 rpm for 72 h: *A. f*, conventional *A. ferrooxidans*; *A. f*+EPS, *A. ferrooxidans* with EPS exfoliated but not removed; *A. f*−EPS, *A. ferrooxidans* with EPS exfoliated and removed. Experimental conditions: initial pH = 3.0; FeSO_4_·7H_2_O dosage 11.12 g; *A. ferrooxidans* density 6.0 × 10^6^ cell·mL^−1^.

It can be seen from the SEM images that at the same Fe^2+^ oxidation efficiency, both the biologically and chemically synthesized schwertmannite samples featured agglomeration structures with similar mineral particle sizes (Figure 3). The biologically synthesized schwertmannite had a distinct needle-like burr structure, however had a larger specific surface area than the chemically synthesized schwertmannite. Chan et al. [25,26,27] stated that the EPS of iron bacteria can play a certain role in regulating the morphology and structure of biogenic secondary iron minerals (e.g., the EPS can be used as a template for the concentration, aggregation, and mineralization of ions to form crystal cores). The specific structure of *A. ferrooxidans*-mediated synthesized schwertmannite may therefore be determined by the EPS attached to cell surfaces. As shown in Figure 5a, when the EPS of *A. ferrooxidans* was peeled off after high-speed centrifugation (20,000 g), the EPS content in the supernatant of the *A. f*, *A. f*+EPS, and *A. f*−EPS treatment was 0.16, 1.67, and 0.08 mg·mL^−1^, respectively. Note that the oxidative activity of *A. ferrooxidans* did not change before or after EPS exfoliation, and the curve trend of Fe^2+^ oxidation with time (Figure 5b) was consistent with that of Figure 1a.

**Figure 6:**
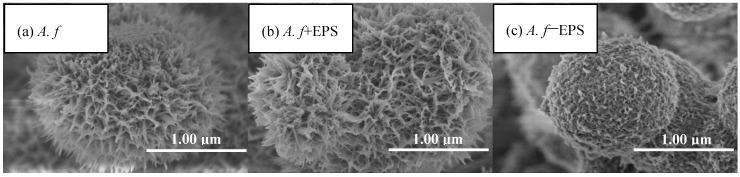
SEM images of biosynthesized schwertmannite under different treatment at 28 °C and 180 rpm for 72 h: (**a**) *A. f*: conventional *A. ferrooxidans*; (**b**) *A. f*+EPS: *A. ferrooxidans* with EPS exfoliated but not removed; (**c**) *A. f*−EPS: *A. ferrooxidans* with EPS exfoliated and removed. Experimental conditions: initial pH = 3.0; FeSO_4_·7H_2_O dosage 11.12 g; *A. ferrooxidans* density 6.0 × 10^6^ cell·mL^−1^.

Figure 6 shows the SEM images of the bio-synthesized schwertmannite before and after the stripping of the EPS from *A. ferrooxidans*. Whether the EPS was attached to the *A. ferrooxidans* surface (*A. f* treatment) or was resuspended in the solution after stripping (*A. f*+EPS treatment) appears to have had no significant effect on the morphology of the schwertmannite. The particles all exhibited “chestnut shell” and “sea urchin” shapes, and all had specific surface areas in the range of 56.76–68.23 m^2^·g^−1^ (Table 1). Interestingly, when the EPS was removed from *A. ferrooxidans* by high-speed centrifugation (*A. f*−EPS treatment), the synthesized schwertmannite particles displayed “wool ball” and “sponge” shapes, and their surfaces were rough (possibly due to the incomplete stripping of the EPS) and contained large numbers of microporous structures. The particle median diameter of the *A. f*−EPS treatment particles was determined to be ~1.21 μm, and the specific surface area was 86.43 m^2^·g^−1^, far larger than those under the other two treatments. Though it has been reported that EPS can regulate the appearance features of biogenetic minerals, excessive needle-like burr structures are likely to cover or block schwertmannite’s pores. We speculate that this may reduce the specific surface area of the particles, thereby affecting their ability to undergo ion adsorption or exchange.

**Figure 7:**
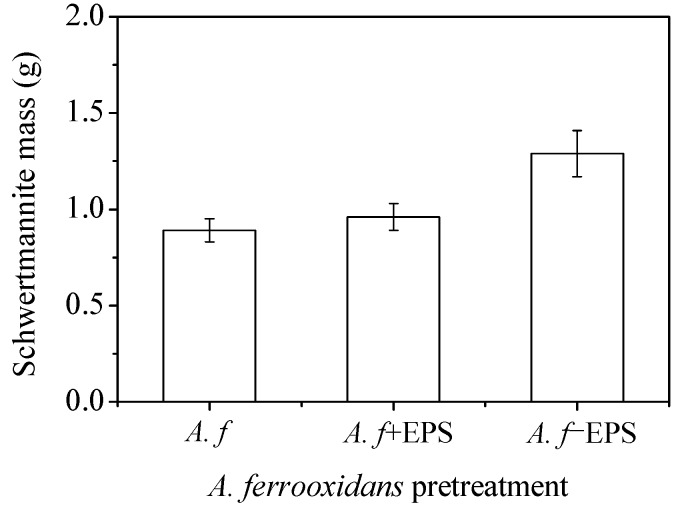
The mass of the biosynthesized schwertmannite under different treatments at 28 °C and 180 rpm for 72 h: *A. f*, conventional *A. ferrooxidans*; *A. f*+EPS, *A. ferrooxidans* with EPS exfoliated but not removed; *A. f*−EPS, *A. ferrooxidans* with EPS exfoliated and removed. Experimental conditions: initial pH = 3.0; FeSO_4_·7H_2_O dosage 11.12 g; *A. ferrooxidans* density 6.0 × 10^6^ cell·mL^−1^.

Figure 7 shows the yields of the *A. ferrooxidans*-synthesized schwertmannite before and after the stripping of the EPS. Although the Fe^2+^ oxidizing ability of *A. ferrooxidans* did not change significantly before and after EPS stripping (Figure 5b), the yield of the minerals synthesized by *A. ferrooxidans* after the stripping of the EPS was much higher than that of the other two treatments (the yields of *A. f*, *A. f*+EPS, and *A. f*−EPS treated minerals were 0.89, 0.96, and 1.29 g, respectively). Therefore, it can be inferred that, compared with the chemical method, the fundamental reason that the biological method had a lower Fe precipitation efficiency and schwertmannite yield is that the secretion of the EPS by *A. ferrooxidans* had a certain inhibitory effect on mineral formation.

In addition, the Fe/S molar ratio is also a very important parameter for characterizing iron hydroxysulfate minerals. According to Bigham et al. [36], the ideal schwertmannite, Fe_8_O_8_(OH)_6_(SO_4_), has an Fe/S molar ratio of 8. In this study, elemental analysis revealed that the Fe/S molar ratios of schwertmannite via the *A. f*, *A. f*+EPS, and *A. f*−EPS treatments were 6.15, 5.92, and 4.87, respectively. The corresponding chemical formulas could be expressed as Fe_8_O_8_(OH)_5.24_(SO_4_)_1.30_, Fe_8_O_8_(OH)_5.19_(SO_4_)_1.35_, and Fe_8_O_8_(OH)_4.23_(SO_4_)_1.64_ (Table 1). This result indirectly indicates that the stripping of the EPS from the surface of *A. ferrooxidans* can increase the incorporation of SO_4_^2−^ in the synthesis of schwertmannite, which may also provide a basis for ligand exchange, adsorption, and fixation of CrO_4_^2−^ and AsO_4_^3−^ [2]. Generally speaking, the physicochemical properties of schwertmannite synthesized by *A.f*−EPS treatment are superior to those of the other two treatments in terms of particle median diameter, specific surface area, and Fe/S molar ratio. The mechanism through which the EPS affects the properties of the secondary iron minerals is still unclear, however, and should be studied further in subsequent experiments.

## 4. Conclusions

This study is the first to investigate the differences between the chemical (H_2_O_2_-FeSO_4_) and biological methods (*A. ferrooxidans*-FeSO_4_) for the synthesis of schwertmannite at the same Fe^2+^ oxidation efficiency. The results indicate that the chemical method is more conducive to the synthesis of schwertmannite than the biological method. The biosynthesized schwertmannite displayed “chestnut shell” or “sea urchin” particle shapes, with a particle median diameter of ~1.52 μm and a specific surface area of 58.79 m^2^·g^−1^. In contrast, the surface of the chemically synthesized schwertmannite was relatively smooth, with a particle median diameter of ~1.34 μm and a specific surface area of only 6.31 m^2^·g^−1^. In other words, the synthesis of schwertmannite is not only affected by the efficiency of Fe^2+^ oxidation, but also by the characteristics of *A. ferrooxidans*. After the stripping of the EPS from *A. ferrooxidans* by high-speed centrifugation, the biomineralized schwertmannite changed from “chestnut shell” or “sea urchin” to “wool ball” or “sponge” particle shapes, with a larger specific surface area of 86.43 m^2^·g^−1^. Meanwhile, the yield of schwertmannite catalytically synthesized by *A. ferrooxidans* increased by about 45% compared with that before stripping. A new discovery can be inferred from this study: the fundamental reason for the lower Fe precipitation efficiency and schwertmannite yield of the biological method is that the secretion of EPS by *A. ferrooxidans* has a certain inhibitory effect on mineral formation.

## Figures and Tables

**Table 1:** Physicochemical properties of biosynthesized schwertmannite under different treatments at 28 °C and 180 rpm for 72 h: *A. f*, conventional *A. ferrooxidans*; *A. f*+EPS, *A. ferrooxidans* with EPS exfoliated but not removed; *A. f*−EPS, *A. ferrooxidans* with EPS exfoliated and removed. Experimental conditions: initial pH = 3.0; FeSO_4_·7H_2_O dosage 11.12 g; *A. ferrooxidans* density 6.0 × 10^6^ cell·mL^−1^.

Treatment	Median Diameter (*d_50_*) (μm)	Specific Surface Area (m^2^·g^−1^)	n(Fe)/n(S)	Mineral Chemical Formula
*A. f*	1.62	68.23	6.15	Fe_8_O_8_(OH)_5.24_(SO_4_)_1.30_
*A. f*+EPS	1.65	56.76	5.92	Fe_8_O_8_(OH)_5.19_(SO_4_)_1.35_
*A. f*−*EPS*	1.21	86.43	4.87	Fe_8_O_8_(OH)_4.23_(SO_4_)_1.64_
